# Exploring autonomic modulation: day-to-day recovery after exercise sessions in breast cancer survivors

**DOI:** 10.3389/fonc.2023.1231683

**Published:** 2023-08-08

**Authors:** Ana Myriam Lavín-Pérez, Daniel Collado-Mateo, Carmen Hinojo González, Ana de Juan Ferré, Cristina Ruisánchez Villar, Xián Mayo, Alfonso Jiménez

**Affiliations:** ^1^ Sport Sciences Research Centre, Rey Juan Carlos University, Madrid, Spain; ^2^ GO Fit LAB, GO Fit Life, Science and Technology, S.A., Madrid, Spain; ^3^ Program of Epidemiology and Public Health (Interuniversity), Ph.D. International School Rey Juan Carlos University, Madrid, Spain; ^4^ Oncology Department, Hospital Universitario Marques de Valdecilla, Santander, Spain; ^5^ Instituto de Investigación Marqués de Valdecilla (IDIVAL), Santander, Spain; ^6^ Cardiology Department, Hospital Universitario Marques de Valdecilla, Santander, Spain; ^7^ Advanced Wellbeing Research Centre, College of Health, Wellbeing and Life Sciences, Sheffield Hallam University, Sheffield, United Kingdom

**Keywords:** exercise therapy, oncology, long-term recovery, heart rate variability, lnRMSSD, lnSDNN, autonomic nervous system

## Abstract

**Purpose:**

The current study aimed to analyze the changes in heart rate variability (HRV) 24h, 48h and 72h after exercise sessions in breast cancer survivors.

**Methods:**

Sixteen survivors who had undergone chemotherapy and radiotherapy were included. Participants trained resistance and cardiovascular components 3 times per week. The intervention was supervised and delivered online for 4 weeks. In this period, patients measured their HRV daily obtaining the lnrMSSD and lnSDNN values of: day 0 (the morning of the training sessions), 24h, 48h and 72h after exercise.

**Results:**

Significant changes in lnrMSSD (p=0.015) and lnSDNN (p=0.031) during recovery times and lnSDNN during the weeks were found (p=0.015). The most prominent differences were identified between the baseline measurement taken on day 0 and 24h after exercise (p=0.007 and p=0.048, respectively) and between measurements obtained 24h and 48h after the training session (p=0.019 and p=0.026, respectively).

**Conclusion:**

Our study suggests that patients may decrease their lnrMSSD and lnSDNN values 24h after exercise and they were close to recover 48h after the sessions. In this regard, HRV may be an useful tool to monitor their recovery and exercise tolerance.

## 1 Introduction

Advancements in adjuvant therapy have resulted in enhanced survival rates among breast cancer patients (1). However, these advances have also led to an increased risk and longer duration of long-term side effects of both the disease and surgery, radiation therapy, chemotherapy, and endocrine consequences (2). Among the side effects of treatments, the development of toxicity and its associated morbidities, such as cardiovascular diseases (3), imbalances in body composition (4), chronic pain and fatigue (5), and low levels of cardiorespiratory capacity (6), strength, and quality of life of the patients are the most alarming. To address this issue, exercise therapies are being developed to mitigate these adverse effects, to recover or, at least, maintain the affected parameters, thereby improving the life expectancy, physical function, or health-related quality of life, as well as reducing cancer-related fatigue of both patients and survivors (7).

Furthermore, exercise can positively influence the autonomic modulation in cancer patients. Heart rate variability (HRV) has emerged as a noninvasive measure of autonomic nervous system (ANS) function in breast cancer (8). HRV is a measure of the variation in time between successive heartbeats, which reflects the activity of the ANS (9). Specifically, HRV is altered as a consequence of cancer side effects (10) exhibiting, for instance, significantly lower levels of parasympathetic activity and overall HRV, compared to healthy individuals (11). In this regard, previous studies have shown that exercise can increase the root mean square of successive differences between normal heartbeats (RMSSD) and the Standard deviation of interbeat intervals from which artifacts have been removed (SDNN). More specifically, exercise may benefit patients by decreasing the overactivation of the sympathetic nervous system (SNS) and increasing the enhancement of the parasympathetic nervous system (PNS) improving, in this way, the modulation imbalance (12, 13).

Although there is evidence supporting the chronic benefits of exercise on HRV (14), no studies have investigated the acute day-to-day variations of HRV during exercise interventions in cancer patients. In contrast, in high-performance athletes and healthy populations, HRV can proportionate objective measurements about how individuals can recover after exercise sessions (15) so it has the potential of monitoring the physiological stress or potential maladaptive states (16). Within exercise acute doses, RMSSD and high-frequency (HF) measures have been shown to decline in healthy people 24h after a high-intensity session, in contrast to endurance training. This would indicate a suppression in parasympathetic activity, which may be recovered at 48h (17) depending on the exercise volume (18) and the fitness level of the athlete (19). In addition, some investigations have found that athletes can take up to 48 hours for full recovery to occur, and in some cases, there may be an “overshoot” above pre-exercise levels prior to the 48h measure (20, 21). However, when the training session involves intense training, RMSSD could still be reduced 48h after exercising (22, 23) and with resistance training variables such as HF, very low frequency and mean-variance decline significantly within 24 hours of the session and reverted 72h after to baseline values (24).

Therefore, HRV is commonly used in athletes to monitor and control training effects and adaptations, but little is known about its applicability in the rehabilitation of chronic patients. Given the side effects of cancer treatments, such as chemotherapy-induced cardiotoxicity and radiation-induced damage to the heart, monitoring the time needed to recover HRV variables after exercise sessions could be crucial to understand the adequate adaptation to exercise and the long-term recovery of cancer patients. Monitoring the 24h, 48h and 72h after exercise recovery can provide useful information to prescribe adequate exercise volume or rest in breast cancer patients, as happened with athletes and healthy populations (16). In this regard, the purpose of the current study is to analyze the changes in HRV 24h, 48h and 72h after exercise sessions in breast cancer survivors who have received chemotherapy and radiotherapy.

## 2 Methods

The study design adhered to the Helsinki Declaration of 2014 (25). It involved a 4-week physical exercise intervention where women with breast cancer underwent an initial evaluation and daily measurements of HRV.

### 2.1 Participants

The present study was conducted with a total of 16 participants recruited exclusively from the Hospital Universitario Marqués de Valdecilla (Spain). The patient selection process followed a set of specific inclusion criteria, which comprised of being a female between the ages of 18 and 65, having a history of breast cancer, and having completed chemotherapy and radiotherapy between one to five months before the beginning of the study. The exclusion criteria for patients included any injury or motor problem that would restrict exercise, development of any other cancer type during the intervention, a scheduled operation before inclusion and a heart problem that was not compatible with the exercise program, as determined by an echocardiogram before the start of the intervention.

Moreover, prior to conducting the study, the sample size was calculated utilizing G*Power software (3.1.9.5) (26). Since this is the first study conducted in breast cancer patients, data reported in the study by Hautala, Tulppo (20) was employed. Specifically, means and SD before and 24 after the exercise stimulus from a single group were taken as reference. Considering the effect size from that study and assuming an alpha error probability of 0.05, and a power (1-beta error probability) of 0.85, a total of 16 participants were needed.

The study conformed to ethical standards approved by the ethical committees of Rey Juan Carlos University (approval number: 1901202103121) and Valdecilla Health Research Institute (IDIVAL) at the University Hospital of Marqués de Valdecilla (register identification number: 2021.214). The recruitment was performed by the medical team who selected patients following the mentioned criteria once they have finished the radiotherapy process at the hospital. Participants were informed by the oncology group and the exercise professional delivering the intervention of the pertinent details of the study, including potential benefits and risks associated with exercise.

### 2.2 Exercise intervention

The duration of the intervention was 4 weeks but contact with the patients started approximately one month before. Before the start of the evaluation and experimental phase, all participants were asked to maintain their usual lifestyle and continue taking any medications they were already taking throughout the trial.

The exercise intervention was designed by the research group and carried out by one of the researchers and exercise professionals specialized in training and conditioning for oncology patients. The training sessions were home-based, all sessions were delivered online and supervised by the trainer. Moreover, to ensure safety, control and adherence to the exercise prescription, patients’ heart rate (HR) was monitored and visualized by the professional and the patient in real time. For this purpose, patients were given a MyZone Chest band and a Training kit (including bar and discs kit) at the face-to-face initial evaluation. Participants trained three times per week for approximately 65 minutes. Each session involved a warm-up of ≈10 minutes, a principal section of ≈ 50 minutes and a cool-down of 5 minutes. The warm-up was performed to move the joints involved in the training and activate the abdominal and lumbopelvic musculature of the patients and the cool-down included mobility and stretching of the musculature exercised.

The principal section of the sessions included strength (≈ 38 min) and cardiovascular training (≈ 12 min). As **Table 1** shows different cardiovascular exercises were proposed depending on the needs of each patient, both for possible biomechanical limitations or considering the best fit for achieving the prescribed intensity. The identification of reserve heart rate values to prescribe intensity was carried out through an initial evaluation where each patient’s resting heart rate was measured. The maximum heart rate was estimated by evaluating the theoretical maximum and the maximum achieved in the Modified Bruce Test (27). The adjustment of strength loads was carried out by evaluating the maximum force of each patient with a lineal encoder in the initial evaluation testing the 1RM of different exercises (Chronojump, Barcelona, Spain).

**Table 1:** Description of the exercise intervention.

Warm-up	Principal part	Cool-down
-Articular mobility - Abdomen and gluteus activation	*Cardiovascular training:* 2 sets (4 intervals x 1 min x 30s rest) - 65-80% HRreserve: walking while raising knees, walking while stretching arms, running in place, and step touch with wide arms. *Resistance training*: 4 sets (8-10 repetitions) x 1 min rest - 55-70% (1RM): (a) Incline raw, chair squat, arm curl, glute bridge (b) Lunge in place, arm extension, chair squat and chest press.	- Stretching of the muscles worked - Chest and arm-specific stretchings

HRr, reserve heart rate; 1RM, one-repetition maximum.

### 2.3 Measurements

As mentioned above, an initial face-to-face evaluation of the patients was carried out before starting the intervention. On the one hand, an echocardiogram was performed at the hospital to detect anomalies that were incompatible with the exercise program. Additionally, resting heart rate, maximum heart rate achieved during the Modified Bruce submaximal test and the 1RM of the back squat, lunge, deadlift, biceps and incline raw were measured to correctly prescribe in the exercise intervention. During this assessment process, clinical and demographic information about the patients was also obtained. Specifically, the following data was collected: age of the patients, type of breast cancer, chemotherapy treatment received, treatment received during the intervention, and time elapsed since completion of radiotherapy. Additionally, systolic and diastolic blood pressure data was taken from the screening echocardiogram evaluation.

#### 2.3.1 Heart rate variability measurements

The data for photoplethysmography-based HRV analysis were collected using a smartphone and the HRV4Training app, which has been previously validated (28) and used in multiple research studies to analyze exercise recovery and physiological stress (29). The processing steps involved averaging the red, green, and blue channels over the entire frame before converting to the HSV (Hue, Saturation, Value) color space (30). The intensity component of the signal was then filtered using a Butterworth bandpass filter with a frequency pass band between 0.1 and 10 to remove noise and the DC component of the signal while preserving the AC component. The signal was up-sampled between 30 and 180 Hz using cubic spline interpolation to increase the resolution of HRV feature computation. A slope inversion algorithm was used for peak detection, and peak-to-peak intervals were corrected for artifacts by removing intervals that differed from the previous interval by more than 20% (28, 30).

Patients’ daily HRV was autonomously measured during the 4-week exercise program. To ensure that all patients were proficient in using the mobile app, it was installed during the initial face-to-face physical assessments, carried out for the establishment of % load and intensity. Patients were trained in how to use the app, and their device was checked for compatibility to ensure quality measurements. A period of one week of familiarization with the app was also provided before the study began, during which the trainer checked daily measurements to provide feedback or assistance as needed. Additionally, a visual explanatory manual was provided to ensure the correct use of the app.

During the course of the exercise intervention, patients measured their HRV every morning while lying down. Upon awakening, the subjects retrieved their mobile phones and assumed a supine position for 5 minutes. During this time, the smartphone camera was positioned with the flashlight on the left index finger to capture a signal for 1 minute (28). If the rMSSD data were deemed inappropriate due to user error (e.g., finger movement over the camera), the subject was notified and asked to perform a new recording until a successful measurement was obtained. Once feedback indicating a correct measurement was received, the subjects stored the information for automatic transmission and the researcher download directly the data from the Hrv4trauing app (csv.)

### 2.4 Outcomes

From all the patients daily raw data of the 4 weeks were downloaded from the HRV4Training app and the measurements were classified into the following categories: measurements before training, conducted on the morning of the training day (Day 0), measurements on the morning after training (24h post-exercise), measurements on the second morning after training (48h post-exercise) and measurements on the third morning after training (72h post-exercise).

Among the variables collected by the mobile application, rMSSD and SDNN were extrapolated. rMSSD is a measure of the variation in the length of time between successive normal-to-normal heartbeats (31). It is calculated by taking the square root of the average of the squared differences between successive normal-to-normal intervals. RMSSD is often used as an indicator of the activity of the parasympathetic nervous system, which is responsible for regulating the body’s relaxation response (31).

SDNN, on the other hand, is a measure of the overall variability in the length of time between successive heartbeats, including both normal-to-normal and abnormal beats (31). It is calculated by taking the standard deviation of all the normal-to-normal intervals over a given period. SDNN is often used as an indicator of the activity of the sympathetic and parasympathetic nervous systems, which are responsible for regulating the body’s stress and relaxation responses (9, 32).

### 2.5 Statistical analysis

Before conducting the analysis, the neperian logarithm of rMSSD and SDNN was calculated based on recommendations from previous literature to improve distribution and mitigate the influence of extreme values (33). Thus, the main variables of the investigation were lnrMSSD and lnSDNN.

Afterward, as previous HRV investigations have done (32), to facilitate comparison between participants, the recovery value of each variable at different time points was calculated relative to the measurement on day 0. Day 0 represented the value that each participant had on the morning of the training session. For example, the recovery value of lnrMSSD at 24h of week 3 (24h post-exercise in the third week), was obtained by subtracting the average lnrMSSD value of day 0 from the average lnrMSSD value 24h after exercise of that week, dividing the result by the average lnrMSSD value of day 0, and multiplying by 100 (([lnrMSSD_24h_w3] - [[lnrMSSD_day0_w3]])/([@[lnrMSSD_day0_w3]])) *100).

Once these variables were obtained, the IBM SPSS Statistics 28 version software was used for the analysis (34). To show the differences between recovery times, and between weeks a separately repeated measures ANOVA was carried out. Then, to investigate changes in recovery times and changes over weeks, a Bonferroni-adjusted repeated measures ANOVA was used for pairwise comparison of the different interactions. A repeated measures ANOVA was performed for each week separately to examine differences in each recovery time with respect to measurement from day 0, and Bonferroni adjustment was used for pairwise comparisons between recovery times. To investigate differences between weeks, a repeated measures ANOVA was performed for each recovery time separately, and Bonferroni adjustment was used for pairwise comparison between weeks.

Statistical significance was considered when the p-value was ≤ 0.05. Additionally, effect size (partial eta squared, ηp²) was used to determine the proportion of the total variance of the response variable explained by a particular predictor in the model. A partial eta squared value of 0.01 was considered small, 0.06 was moderate, and 0.14 was large for the interpretation of ηp² (35).

## 3 Results

### 3.1 Participants characteristics

A total of 16 patients, with a mean age of 50.13 and a standard deviation of 9.17 participated in the study. Their ages ranged from 30 to 65 years, as can be seen in **Table 2** along with detailed information on each patient. The mean heart rate at rest was 68.85 with a standard deviation of 7.33 beats per minute. Regarding systolic and diastolic blood pressure, the average for the patients was 117.81 (with a standard deviation of 17.34) and 75.19 (with a standard deviation of 11.13), respectively. As detailed in the **Table S1** (**Supplementary Data**), the most common type of breast cancer among the patients was luminal. Specifically, four were treated for type A and nine for type B. These patients, due to the nature of their cancer, received hormonal or aromatase inhibitor treatment during the exercise intervention. The remaining three patients had triple-negative breast cancer.

**Table 2:** Participants characteristics.

Patient	Age	Heart Rate	Blood pressure (S/D)	Treatments during intervention	Patient	Age	Heart Rate	Blood pressure (S/D)	Treatments during intervention
1	54	68	131/84	Tamoxifen	9	59	73	110/70	Letrozole
2	44	66	97/65	Tamoxifen	10	46	66	94/57	Tamoxifen
3	58	71	120/86	Letrozole	11	59	69	160/99	Letrozole
4	42	75	120/80	Tamoxifen and Zoladex	12	65	68	110/76	None
5	46	65	106/66	None	13	51	62	128/80	Tamoxifen
6	49	68	100/70	Zoladex and exemestane	14	51	75	125/80	Letrozole
7	62	73	144/85	Letrozole	15	40	67	120/80	Tamoxifen
8	46	75	110/65	Tamoxifen	16	38	62	110/60	None

S, Systolic blood pressure; D, Diastolic blood pressure.

### 3.2 Acute effects of exercise on heart rate variability

**Table 3** shows participants’ global absolute results (rMSSD and SDNN) in means and standard deviations. The differences between recovery times are shown in **Table 3** where significant differences were achieved in both variables (rMSSD p-value = 0.033; SDNN p-value = 0.025). Whereas there were no significant differences in any of the variables (rMSSD p-value= 0.638; SDNN p-value = 0.335.

**Table 3:** Means and standard deviations of participants’ lnrMSSD and lnSDNN (n=16).

Recovery times	rMSSD	SDNN	Intervention weeks	rMSSD	SDNN	
Day 0	54.85 ± 38.92	59.79 ± 34.36	Week 1	47.33 ± 28.85	52.01 ± 26.71	
24h post	44.33 ± 25.91	48.93 ± 24.78	Week 2	51.45 ± 26.77	55.81 ± 24.22	
48h post	51.15 ± 32.75	56.93 ± 31.34	Week 3	52.01± 26.67	57.31 ± 25.17	
72h post	55.25 ± 27.99	58.87 ± 23.46	Week 4	54.78 ± 37.66	59.39 ± 32.96	
p-value	p=0.033a	p=0.025a	p-value	p=0.638b	p=0.335b	

rMSSD, root mean square of successive differences between normal heartbeats; SDNN, standard deviation of interbeat intervals from which artifacts have been removed; a: p-value of the repeated measures ANOVAs of the differences between the recovery times; b: p-value of the repeated measures ANOVAs of the differences between weeks.

#### 3.2.1 Differences between recovery exercise times

**Figure 1:**
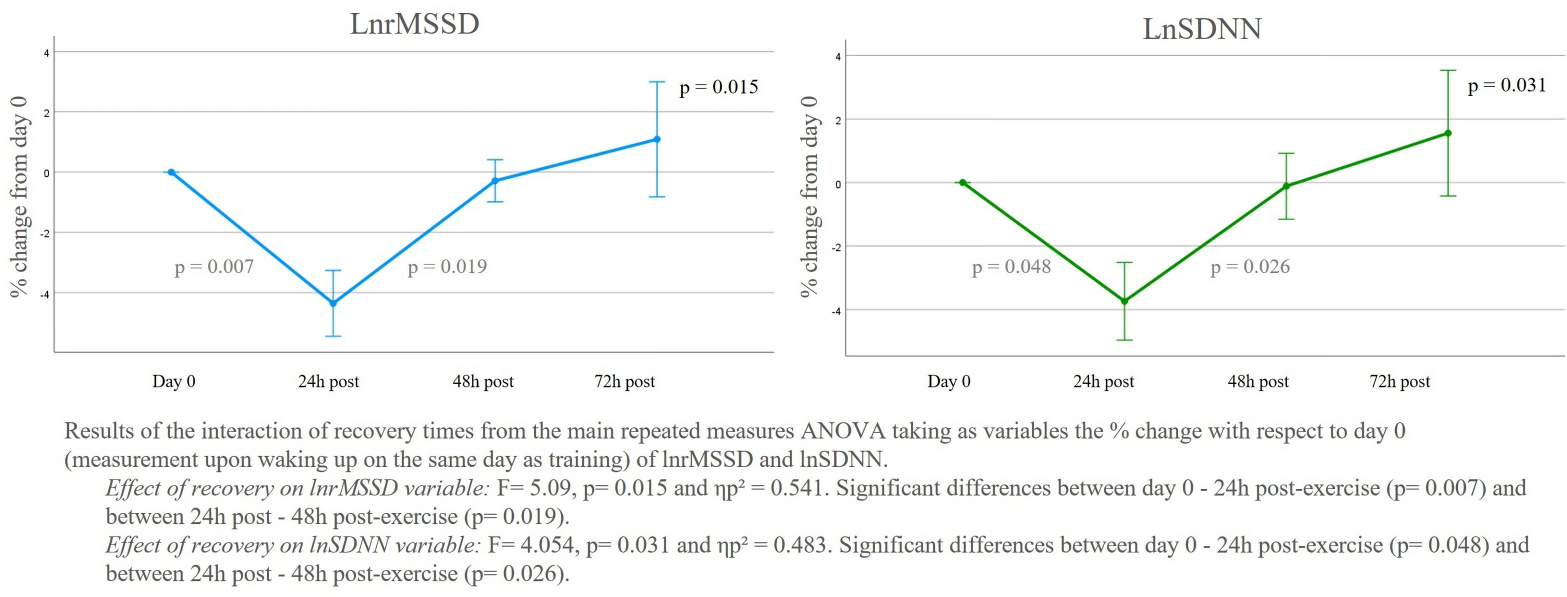
LnrMSSD and lnSDNN % of change from day 0 of each week and recovery time.

In order to extensively explore the interaction between the recovery times and the weeks, the following analyses will include the study of the variables utilizing percentage change. As shown in **Figure 1**, the results of the repeated measures ANOVA for lnrMSSD, using the % change with respect to day 0 (measurement upon waking up on the training day) as variable, showed a significant effect between recovery times (F= 5.09, p= 0.015 and ηp² = 0.541). Pairwise comparisons demonstrated significant differences between Day 0 and 24h post-exercise (p=0.007), with a mean difference of |4.357| and a standard error of 1.094, and between 24h and 48h post-exercise (p=0.019), with a mean difference of |4.071| and a deviation of 1.126. All other pairwise comparisons had a p-value of 1.000, except for the 24h and 72h interaction with a p-value of 0.099.

A similar trend was observed in lnSDNN, where a significant effect between recovery times was also found (F= 4.054, p= 0.031 and ηp² = 0.483). In this global analysis regarding the average of all weeks, significant differences were reached between Day 0 and 24h post-exercise (p=0.048), with a mean difference of |3.737| and a standard error of 1.22, and between 24h and 48h post-exercise (p=0.026), with a mean difference of |3.622| and a standard error of 1.080 (**Figure 1**). The rest of the comparisons were not significant.

#### 3.2.2 Differences between the recovery measures of each training week

**Figure 2:**
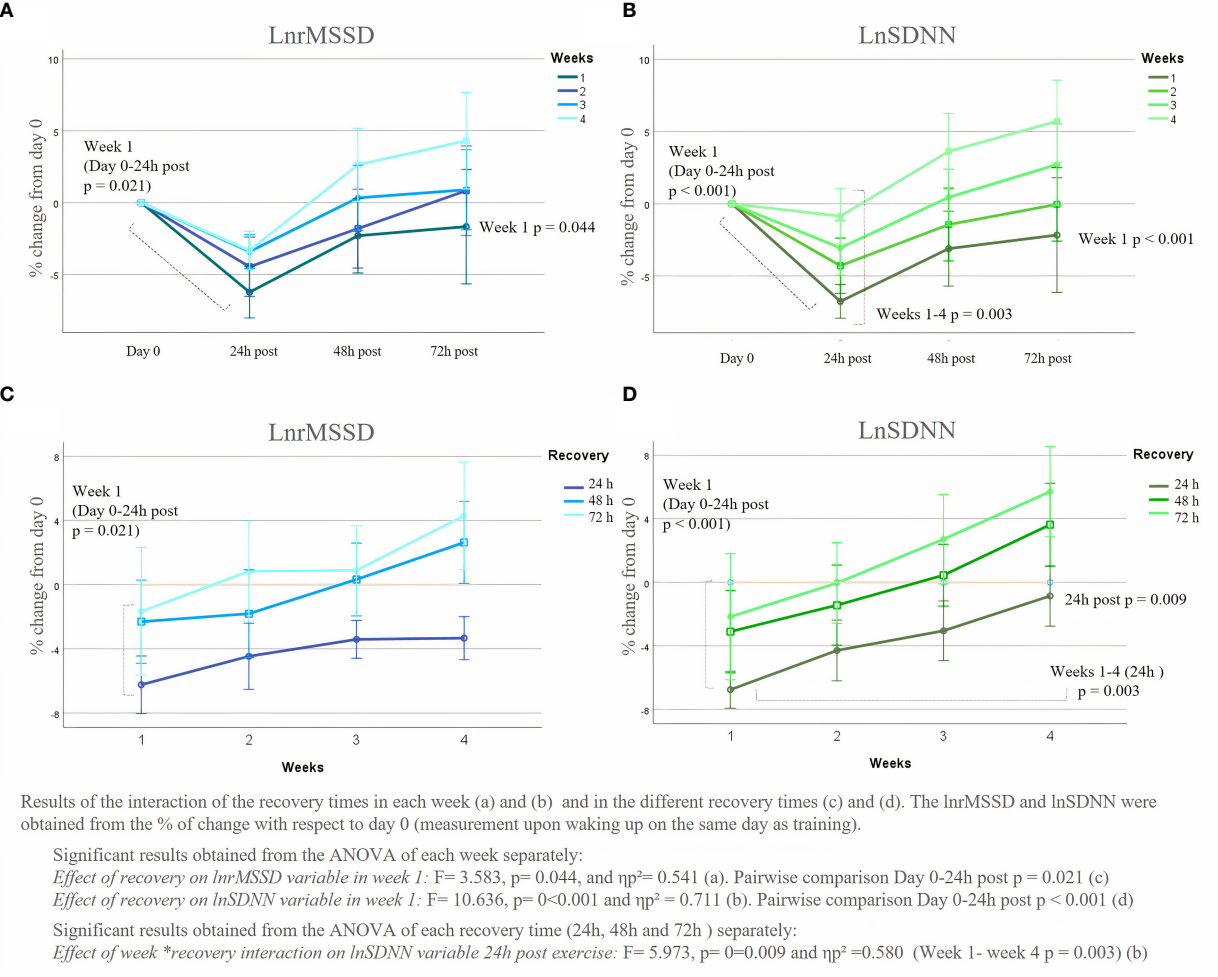
LnrMSSD and lnSDNN % of change of the average recovery times (24h, 48h and 72h post-exercise) from day 0.

The analysis of lnrMSSD variable in each week is shown in **Figure 2**. Significant differences in recovery times were only found in week 1, with a value of F= 3.583, p= 0.044, and ηp² = 0.541. Specifically, this significance, in the pairwise analysis, showed significant differences between the measurement on day 0 (training day) and 24h post-exercise. In the remaining weeks, although week 4 showed a tendency to interact, there were no significant differences (Week 2: F= 2.088, p= 0.151, ηp² = 0.325; Week 3: F= 2.796, p= 0.082, ηp² = 0.392; Week 4: F= 3.201, p= 0.059, ηp² = 0.425).

In lnSDNN variable, similar results were obtained in the pairwise comparison of the principal ANOVA, with significant results between Day 0 and 24h post-exercise in week 1 (p<0.001). The ANOVA performer of each week showed that the F value of week 1 was 10.635 and a ηp² value of 0.711, whereas, within the rest of the weeks, no significant differences were found between the recovery times (Week 2: F = 2.283, p = 0.127, ηp² = 0.345, Week 3: fonc.2023.1231683F = 2.7128, p = 0.146, ηp² = 0.329, Week 4: F = 3.000, p = 0.147, ηp² = 0.329).

## 4 Discussion

The current study is the first aimed to analyze the changes in HRV 24h, 48h and 72h after exercise sessions in breast cancer patients who have received chemotherapy and radiotherapy. The application of HRV as a tool to track and monitor exercise acute recovery has undergone investigation in the athletic population in the last decade (14). However, findings from sports performance have not been tested in other populations, such as breast cancer patients. Although most research supports the benefits of regular physical exercise on HRV through pre- and post-intervention analyses in cancer patients (14), to our knowledge, no article has analyzed the daily oscillations and adaptations of patients to every session, which could be extremely important to understand chronic improvements in HRV caused by physical exercise.

The main finding of the current study was that patients significantly changed lnrMSSD and lnSDNN during the recovery times and lnSDNN over the weeks. Notably, the most prominent differences were observed between the baseline measurement, taken on day 0 of the training sessions, and the measurement taken the morning after the training session (24h after). Additionally, another notable change was observed between the measurement taken the morning after the training session (24h) and the measurement taken 48 hours after the training session, which may be related to a successful adaptation to the stimulus of exercise. Results from the current study suggest that HRV may be a useful tool to evaluate the daily adaptations to exercise of patients and, consequently, to design and adapt the exercise training to individual responses. Although these results are promising, future studies should deeply explore the sensitivity and usefulness of HRV to daily design exercise interventions.

As can be seen from the results obtained, both lnrMSSD and lnSDNN decreased significantly in the measurements taken the morning after exercise (24h). These decreases are explained by the acute stress that can be caused by the dose of exercise and the lack of recovery of the body from it at 24h. Although it has not been previously studied in people with breast cancer, this reaction has also been found in athletes and healthy people when after practicing high-intensity exercise or strength training (17, 24). In this regard, beginners, as happens with the population of the current study, experience higher sympathetic activation in response to acute exercise sessions compared to more experienced ones. When the body faces, acutely, a strong stress stimulus, the SNS becomes overactive, releasing catecholamines such as norepinephrine and dopamine, and the PNS decreases dramatically (36). Thus, variables such as lnrMSSD (linked to the PNS) and lnSDNN (the combination of both SNS and PNS) decrease (14). In fact, as can be seen in **Figure 2**, the lnrMSSD variable tended to decrease more than lnSDNN.

Previous studies suggested that stress effects could be harmful to patients since the release of chronic catecholamines and cortisol could favor the appearance and progression of breast cancer. Indeed, higher levels of epinephrine and norepinephrine have been detected in fluids and tissues of cancer patients, suggesting that catecholamines and adrenergic signaling are involved in cancer pathogenesis (37, 38). However, studies in murine models have shown that catecholamines released by exercise do not promote tumor growth, on the opposite, they seem to have tumor growth suppression effects (39, 40).

Breast cancer survivors experience, after chemotherapy and radiation therapy administration, the parasympathetic nervous system more suppressed (12), which may result in even greater decreases at 24 h than in healthy or athletes population, as happened in rest conditions. In contrast, highly trained athletes experience a faster parasympathetic reactivation without decreasing lnrMSSD levels as much at 24 hours (19). In this regard, the findings of the sub-analysis performed in the current study obtaining, in the measurements of lnrMSSD and lnSDNN significant differences between day 0 and 24 hours after the training session only during week 1 can be understood. Therefore, following workout sessions would stimulate the sympathetic system less while maintaining the same level of effort, which can lead to quicker recovery. Nevertheless, in the current study, patients significantly increased their lnrMSSD and lnSDNN at 48h after exercise compared to after 24h, but without reaching the values of day 0 (before exercise). Actually, the day 0 measurements were not reached until 72h after the exercise dose, which is a phenomenon that occurs more rapidly in any kind of athlete and trained individuals, indicating a faster recovery to exercise (22).

Interestingly, unlike what is sought in athletes when receiving a very demanding stimulus (41), it seems that in breast cancer survivors the overshoot at 48h does not occur. This phenomenon of parasympathetic rebound, shown in athletes’ investigations, has been linked to an increase in vagal activity, the parasympathetic modulation of heart rate. The difference perceived in the current results may be due to the mentioned autonomic imbalance still present in untrained women or due to the exercise doses given was not so exhausting. However, our data show a trend for values at 72 hours to exceed those of day 0 as they become more trained, from week 3 onwards, and even at 48h in week 4. However, this is only a speculation to be analyzed in future studies since with just 4 weeks of training these changes are not fully perceived. The analysis of a longer program would provide this information since over the course of the 4 weeks of intervention the data from the measurements at 24h have managed to be significantly different between week 1 and week 4 in the lnSDNN variable.

The utilization of new technologies to monitor exercise response, such as HRV has been used in the present study, can be a great help in monitoring physical activity accurately, individualizing exercise prescriptions, providing real-time feedback, and offering reminders, among other benefits. Technology can also facilitate connections between healthcare professionals and patients and allow for the sharing of information with peers increasing interventions adherence (42). In this regard, the intervention in the present study was carried out completely online, as have other studies with oncology patients (43), but with the novelty that the sessions were supervised, and heart rate was monitored live by employing MyZone mobile application with a chest band (44). However, it is important to consider that some individuals may refuse technology, so its selection must be in accordance with the participants’ technological knowledge and program budget, to provide, in this way an exercise facilitator and not a barrier (45). Incorporating technology into exercise programs may be a suitable option provided that the participants are willing to use it and the devices and software align with their needs.

While the present research has yielded interesting findings for the first time, some important limitations need to be considered for future research. Firstly, the sample size of the patient cohort was not very high, which may have limited the ability to draw robust conclusions and obtain more statistically significant results. Additionally, participants’ characteristics were diverse, despite controlling variables such as chemotherapy and radiotherapy treatments. Participants were under different hormone treatments during the study, which may have introduced confounding variables. Furthermore, the age range of participants was broad, leading to heterogeneity in HRV values. While statistical adjustments were made to minimize these differences, it is possible that greater homogeneity could be achieved by further restricting the age range or baseline HRV values for lnrMSSD and lnSDNN variables. Finally, given the lack of studies on this topic in breast cancer patients, caution is advised when interpreting the results. Further studies should consider expand sample size to consider the effect of different treatment regimens and include other parameters of neurovascular modulation of heart rate, as well as test the association of these variables with clinical outcomes in oncology.

## 5 Conclusions

The study investigated the impact of exercise acute recovery on heart rate variability (HRV) in breast cancer patients who had received chemotherapy and radiotherapy. It seems that there are differences in HRV between the recovery times after exercise and throughout the 4-week intervention. In breast cancer patients after chemotherapy and radiotherapy, exercise appears to reduce parasympathetic activation at 24 hours, approaching pre-exercise session measurements at 48 hours and with significantly higher values than at 24 hours. This decline can be attributed to the acute stress response triggered by exercise, leading to increased sympathetic nervous system activity and reduced parasympathetic nervous system activity. These findings have potential implications for future oncological research on exercise tolerance, tailoring and recovery progression with a novelty tool to monitor daily autonomic modulation as is being done in athletes and healthy populations.

## Data availability statement

The original contributions presented in the study are included in the article/**Supplementary Material**, further inquiries can be directed to the corresponding author/s.

## Author contributions

Conceptualization AL-P, DC-M and XM. Methodology, AL-P, DC-M, CG, AF and AJ. Software, AL-P and DC-M. Validation, DC-M, AL-P, XM and CV. Formal Analysis, AL-P, DC-M and AJ. Investigation, DC-M, CG, AF, CV and AJ. Resources, AL-P, XM and DC-M. Data Curation, AL-P, and DC-M. Writing – Original Draft Preparation, DC-M, AL-P and XM. Writing – Review & Editing, AJ, DC-M, CHG and AL-P. Supervision, AF, CV and XM. Project Administration, CG and AJ. All authors contributed to the article and approved the submitted version.
