# Supplementary material for: Exploring autonomic modulation: day-to-day recovery after exercise sessions in breast cancer survivors
Source: Front Oncol. 2023 Aug 8;13:1231683. doi: 10.3389/fonc.2023.1231683 (PMC10442838; doi:10.3389/fonc.2023.1231683)
Supplement: Supplementary file 1 [file Table_1.pdf]

Supplementary data

Table S1. Participants' breast cancer type and chemotherapy treatment received prior to the study.

Patient	Breast cancer type	Chemotherapy treatment
1	Bilateral infiltrating ductal carcinoma<br>a) cT2N2a G3, ypT1b ypN1 (3+/21), ER (+++) 95%; PR (+,++) 10-20%; Ki-67: 20-25%; Herceptest negative)<br>b) cTbN0 G1 , ypT1b pN0gc; ER (+++) 95 %, PR(+++) 80 %, Ki-67: 5 %, HERCEP-TEST: Negative (+). Luminal phenotype A	Induction (paclitaxel and taxol)
2	Moderately differentiated infiltrating ductal carcinoma (IHQ RE positive (+++) 100%; RP Positive (++) in 50%; Ki-<br>67: Positive 20-25 %; equivocal Herceptest with not amplified). T2N1M0 bifocal. ypT1c ypN1 Mx luminal phenotype A.	Neoadjuvant (taxotere adriblastina cyclophosphamide,docetxel-adriamicinaciclofosfamida)
3	Infiltrating ductal carcinoma, G1, cT3 cN1, HR positive, Ki67 24%, HER2-, ypT2, ypN1a (1+/27). Luminal phenotype B.	Neoadjuvant (Adriamycin - cyclophosphamide and taxol)
4	Infiltrating multicentric carcinoma with medullary features G3 cT1cmN1M0, ypT1ypN1M0. ER+++/RP-, Ki-67: 40%, HER2- Luminal phenotype B.	Neoadjuvant (Adriamycin/Cyclophosphamide and paclitaxel)
5	Infiltrating ductal carcinoma pT1c pN0 Mx, triple negative, Ki-67: 60%.	Neoadjuvant (carboplatin-Abraxane and adriamycin-cyclophosphamide).
6	Infiltrating lobular carcinoma cT3 cN0 M0, RH+/HER2-/Ki67 15-20%, luminal phenotype A.	Neoadjuvant (adriamycin - cyclophosphamide and paclitaxel)
7	Infiltrating lobular carcinoma G2 pT3m pN1mi Mx, luminal phenotype B.	Adjuvant (Taxol and adriamycin - cyclophosphamide)
8	Infiltrating ductal carcinoma, pT1c pN0, luminal B pT1cpN0, G3. RH-positive, Her2-negative	Neoadjuvant (adriamycin - cyclophosphamide)
9	Multicentric carcinoma pT2 pN0i+, luminal A, and ER (+++) 95%, PR (++) 70%, HER2 negative and Ki67 of 15%,<br>And an invasive lobular carcinoma with ER (+++) 95%, PR (++) 80%, HER2 negative and Ki67 10%. Luminal phenotype A.	Adjuvant (Taxol and adriamycin - cyclophosphamide)
10	Infiltrating ductal carcinoma g3, pT1c pN1mi (2/9) Mx (Stage IB) with vascular/lymphatic invasion. ER+/RP+ Ki67: 85%, Her2 -. Luminal phenotype B	Adjuvant (adriamycin - cyclophosphamide and paclitaxel)
11	Infiltrating lobular carcinoma, G2, cT1c cN0, ER+++, RP+++, Ki67 15-20%, Her2 negative, luminal phenotype B.	Adjuvant (adriamycin - cyclophosphamide and paclitaxel)
12	Infiltrating ductal carcinoma, G2, cT2N1, triple negative, Ki 67 89%.	Neoadjuvant (carboplatin-Abraxane and adriamycin-cyclophosphamide)
13	Multicentric infiltrating ductal carcinoma, G2, pT2, pN1(mi), Mx, luminal B phenotype	Adjuvant (Taxol and adriamycin - cyclophosphamide)
14	Infiltrating ductal carcinoma cT4cN1M0, ypT3ypN2aM0, G3, Re and RP positive, Her2 negative, Ki67: 30%, luminal B phenotype	Neoadjuvant (adriamycin - cyclophosphamide and paclitaxel)
15	Bifocal infiltrating ductal carcinoma, luminal B, G2, ER +++ 95 %, RP ++ 60-70 %, Ki67 20-25% in the largest, and Ki67 of 10-15%.	Neoadjuvant (adriamycin - cyclophosphamide and paclitaxel)
16	Differentiated infiltrating carcinoma of medullary features T1-2N0Mx RE/RP/her2 negative, Ki 80%. Triple-negative.	Neoadjuvant (carboplatin-Abraxane and adriamycin-cyclophosphamide)
